# Effects of an oral mucosa protective formulation on chemotherapy- and/or radiotherapy-induced oral mucositis: a prospective study

**DOI:** 10.1186/s12885-021-09107-6

**Published:** 2022-01-21

**Authors:** Takao Ueno, Wakako Yatsuoka, Hiroto Ishiki, Kanako Miyano, Yasuhito Uezono

**Affiliations:** 1 Department of Dentistry, National Cancer Center Hospital, 5-1-1 Tsukiji, Chuo-ku, Tokyo, 104-0045 Japan; 2 Department of Palliative Medicine, National Cancer Center Hospital, 5-1-1 Tsukiji, Chuo-ku, Tokyo, 104-0045 Japan; 3 Division of Cancer Pathophysiology, National Cancer Center Research Institute, 5-1-1 Tsukiji, Chuo-ku, Tokyo, 104-0045 Japan; 4 Supportive and Palliative Care Research Support Office, National Cancer Center Hospital East, 6-5-1 Kashiwanoha, Kashiwa-shi, Chiba, 277-8577 Japan; 5 Department of Pain Control Research, The Jikei University School of Medicine, 3-25-8, Nishi-Shimbashi, Minato-ku, Tokyo, 105-8461 Japan

**Keywords:** Bioadhesive, Breakthrough pain, Deglutition, Hydrogel, Mucositis, Opioid analgesics

## Abstract

**Background:**

Oral mucositis (OM) associated with cancer treatment not only impairs patients’ quality of life but also causes treatment delays or changes. This prospective exploratory study was conducted to evaluate the efficacy of Episil® oral liquid, which is an approved protective formulation for the oral mucosa in patients with OM. The extent of the pain-relieving effect, feeling during use, and adverse events or problems were evaluated.

**Methods:**

In total, 10 Japanese cancer patients with OM receiving chemotherapy, pretreatment therapy for hematopoietic stem cell transplantation, or radiation therapy for head and neck cancer were enrolled.

**Results:**

A numerical rating scale (NRS) was used to assess oral pain intensity due to OM. Compared to baseline, the mean NRS began to decrease at 5 min after using Episil® (7.1 ± 1.4 to 4.6 ± 2.87; *p* = 0.264). A significant decrease was observed in the pain score after using Episil® compared with that before using Episil®, and this effect lasted up to 120 min. The protective effects of Episil® were observed 3–5 min after application. Some patients felt slight soreness or discomfort when applying Episil®. However, this discomfort due to Episil®’s stimulation was within the allowable range and transient. No adverse events were observed in any of the cases.

**Conclusions:**

The results of this prospective study showed that Episil® could be an effective treatment to relieve oral pain in Japanese patients with moderate to severe OM, and this newly approved product might adequately support patients’ oral intake.

**Trial registration:**

University Hospital Medical Information Network Clinical Trials Registry (UMIN-CTR) (UMIN000031921).

## Background

Oral mucositis (OM) is a debilitating side effect frequently observed in patients undergoing high-dose chemotherapy, pretreatment therapy for stem cell transplantation, or in patients with head and neck malignancies undergoing radiation therapy [1, 2]. OM can be highly problematic during treatment as it is extremely painful, causes oral intake reduction due to that oral pain, and can be a route of systemic infections [3–5]. Since OM can lead to malnutrition, dehydration, and infection, it can even cause treatment delay or interruption. In addition, previous reports have shown that OM can be a dose-limiting toxicity [5, 6]. Therefore, OM not only affects patients’ quality of life (QOL), but also their prognosis [5–7].

The management of OM during cancer treatment is difficult. Although the Multinational Association of Supportive Care in Cancer [8], National Comprehensive Cancer Network [9], and European Society for Medical Oncology [10] have provided some recommendations for the management of mucositis, the use of benzydamine, photo-bio-modulation, zinc, and glutamine intake [8] is not covered under the Japanese social insurance system. Therefore, in Japan, this complication is addressed by a trial-and-error approach with minimal evidence and few resources.

One of the most important strategies in managing OM is reducing oral pain. Unrelenting oral pain due to severe OM causes subsequent inability to eat and drink, leading to secondary malnutrition and dehydration. Prolonging this condition makes patients’ performance status poorer and potentially interrupts cancer treatments [3, 11, 12]. Non-steroidal anti-inflammatory drugs (NSAIDs), opioids, and local analgesic therapies have been reported to be effective in this patient population [8]. However, they do not always eliminate oral pain because they are generally ineffective against breakthrough pain caused by swallowing or food contact with ulcerative mucositis lesions.

Some coating agents have been designed to form oral mucosal barriers that reduce irritation and OM pain. Various agents, such as viscous liquid mucoadhesive hydrogels, have been suggested [13–15]; however, they were not been approved for use in Japan until 2018. A medical agent, Episil® oral liquid (Marketing Authorization Holder in Japan: Solasia Pharma K. K., Tokyo, Japan), was the first coating agent approved in April 2018. It is a medical agent, developed by Camurus AB., Lund, Sweden, that uses topical bioadhesive technology to continuously cover and protect the affected area of OM. Camurus AB conducted a Phase IIb clinical trial (Study HS-05–161) in patients with head and neck cancer with radiation-induced stomatitis in 2007 and demonstrated the compound’s pain-relieving effect. Episil® received European Community certification in May 2009 as a Class 1 medical device in the European Union and in September 2011, it received 510 (k) clearance from the United States Food and Drug Administration. As of March 2020, it has been approved in 38 countries, including European countries and the United States.

The European Oral Care in Cancer Group and the United Kingdom Oral Management in Cancer Group recommend Episil® for relieving OM pain.

Although Episil® is highly likely to relieve the pain of mucositis, there are few reports of its use in Japanese patients with OM [16], and its effectiveness, feeling during use, and adverse events in Japanese patients are not well known. Therefore, this prospective study aimed to elucidate the clinical efficacy and feasibility of Episil® use in Japanese patients.

## Methods

### Study design and participants

This was a single-center, single-arm, open-label, prospective study. In total, 10 patients being treated at the National Cancer Center Hospital were enrolled from April 2, 2018, to April 25, 2018. Episil® was applied on the mucositis lesions of eligible patients. It comprises soybean phosphatidylcholine (SPC) and glycerol dioleate (GDO), which are natural lipids, and contains no medicinal ingredients. The lipid components SPC and GDO self-assemble upon contact with moisture and form a thin bioadhesive liquid crystalline film. The film acts as a protective barrier and exerts a pain-relieving effect. It is an extremely simple device: a few drops of Episil®’s solution are dropped into the oral cavity (a sufficient amount that covers the entire oral mucosa can be provided by pressing the pump once or twice) and subsequently spread over the affected area with the tongue or the finger. It reacts with water in the saliva and forms an adhesive protective film on the surface of the ulcer within minutes. It comprises soybean phosphatidylcholine (SPC) and glycerol dioleate (GDO), which are natural lipids, and contains no medicinal ingredients. The lipid components SPC and GDO self-assemble upon contact with moisture and form a thin bioadhesive liquid crystalline film. The film acts as a protective barrier and exerts a pain-relieving effect. Data were collected on pain and other outcomes at baseline and 5, 30, 60, and 120 min after application. Similarly, data on adverse events were collected during Episil® use. This study was conducted in accordance with the Declaration of Helsinki, and the study protocol was reviewed and approved by the National Cancer Center Ethics Committee (Approval Number: 2017–400). Written informed consent was obtained from all individual participants included in the study.

### Eligibility

Eligibility criteria are summarized in Table 1.

**Table 1:** Inclusion and exclusion criteria

Criterion	
Inclusion criteria	
1	Patients aged 20 years or older (at the time of providing informed consent)
2	Patients with oral mucositis due to chemotherapy, radiation therapy, a combination of chemotherapy and radiation therapy (chemoradiotherapy), or pretreatment therapy for hematopoietic stem cell transplantation
3	Patients with a score ≥ 5 when starting Episil®. Oral pain due to oral mucositis (the maximum pain combining continuous pain and breakthrough pain) was assessed using a numerical rating scale (11-like Likert scale from 0 to 10) using the Universal Pain Assessment Tool
4	Patients with good general activity status (Eastern Cooperative Oncology Group [ECOG] Performance Status [PS] Scale: 0–2)
5	Patients who are not allergic to any Episil® oral liquid components (glycerindiolate, soy phosphatidylcholine, ethanol, propylene glycol, polysorbate 80, peppermint oil)
Exclusion criteria	
1	Patients with oral cancer lesions
2	Patients with obvious wounds in the oral cavity caused by conditions other than oral mucositis
3	Patients with primary malignant tumors; patients who have lesions in the central nervous system; patients with metastasis/invasion of the central nervous system; or patients suspected to have these aforementioned conditions
4	Patients who received rescue treatment before starting to use Episil® on the day of Episil® use
5	Patients participating in other clinical trials or studies
6	Lactating, pregnant, or likely pregnant female patients
7	Other patients for whom participation in the study was judged to be difficult at the discretion of the researcher

### Outcomes

The primary outcome of this study was the oral pain NRS score. Secondary outcomes included perception, oral-related functions and adverse events.

Perception and oral-related functions were evaluated using the method used in the clinical study conducted by Camurus (HS-05–160 study). This questionnaire included nine questions. Seven questions were related to the feeling during use as follows: “ease of use of Episil®” (easy to use, a little difficult to use, difficult to use); “the feeling and comfort in the mouth when using Episil®” (good, ordinary, or bad); “the time Episil® takes to form a protective film” (approximately 1–2 min, 3–5 min, 5 min or more); “changes in taste sensation” (none – barely none, a little troublesome, very troublesome); “stimulation of mucous membrane” (none – barely none, a little troublesome, very troublesome); “Uncomfortable feeling” (none – barely none, a little troublesome, very troublesome); and “acceptance and willingness to continue using Episil®” (want to keep using Episil® after this study, do not want to use it anymore). The remaining two questions were about oral-related functions: “Speaking difficulties” and “eating difficulties” (none barely any, a little troublesome, very troublesome).

Oral mucosal damage using the National Cancer Institute-Common Terminology Criteria for Adverse Events (NCI-CTCAE) Version 3.0 (diagnostic findings) [17] and adverse events were recorded regardless of whether they had a causal link to Episil® during the treatment period.

### Intervention

**Fig. 1:**
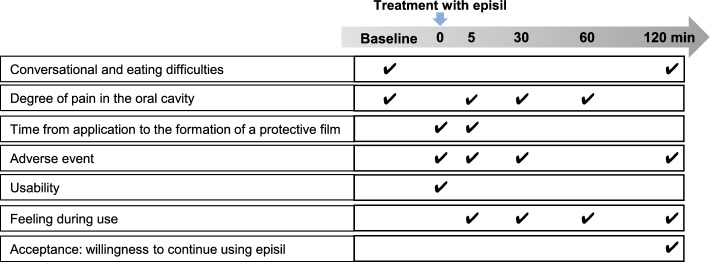
Evaluation time points for various study parameters. Arrow head represent the time points at which data were collected for various study parameters during the evaluation of Episil®

Episil® is in a special container with a pump. The pump was pressed three times to apply Episil® solution to the affected area of the oral cavity. After evaluating the efficacy profile over 120 min following the use of Episil®, continuous use of Episil® was allowed if the patient wished to continue, and the investigator considered it usable and necessary. The use of Episil® was limited to approximately 30 days from the start of its use or until one bottle was used up, whichever was shorter. Adverse events and problems were monitored throughout the period of use (Fig. 1).

During the study period, concomitant analgesic use was allowed. If the patients used any of the following analgesics (acetaminophen, NSAIDs, local anesthetics, or opioids) that may affect the evaluation of oral pain, only regular use of the same dose and frequency, as in the previous study enrollment, was allowed on the first day of using Episil®. If unbearable oral pain developed and the daily doses of these drugs were increased on the day of using Episil®, they were considered rescue treatments.

### Data collection

The oral pain NRS score was evaluated before applying Episil® (baseline), and 5, 30, 60, and 120 min after application. The feeling during use was evaluated 5, 30, 60, and 120 min after applying Episil®. Speaking and eating difficulties were evaluated before Episil® application and 120 min after application. The time until Episil® formed a protective film was evaluated at 1 to 5 min or more after the application of Episil®. Ease of use of Episil® and feeling and comfort in the mouth were evaluated 5 min after Episil® application. Furthermore, 120 min after the application of Episil®, patients were asked whether they wanted to use it repeatedly. Adverse events were evaluated throughout the period of the patients’ use of Episil®. These are summarized in Fig. 1.

### Statistical analysis

Data are represented as mean ± standard deviation for continuous variables or number (frequency) for categorical variables. Oral pain NRS was compared using Friedman’s test, followed by Dunn’s multiple comparisons test. Differences with a *p* ≤ 0.05 were considered statistically significant. Statistical analyses were performed using GraphPad Prism software (v.6.0; GraphPad Software, San Diego, CA, USA) and BellCurve for Excel (Social Survey Research Information Co., Ltd., Tokyo, Japan).

## Results

### Patients’ characteristics

The patients’ characteristics are summarized in Table 2. The study included four males and six females; with a mean age of 61.6 ± 13.6 years; and causes of OM included chemotherapy (six patients), pretreatment therapy for hematopoietic stem cell transplantation (two patients), and radiation therapy for head and neck cancer (two patients).

**Table 2:** Patients’ characteristics and oral mucositis severity

Patient	Age (y)	Sex	PS	Disease	Chemotherapy regimen	Radiation therapy	Concomitant drugs or therapies	Mucositis severity CTCAE v3 (Grade)
**1**	59	M	0	Stomach cancer	Capecitabine	N	Lidocaine gargle	2
**2**	75	M	0	Hard plate cancer	N	Brachytherapy (70 Gy)	Lidocaine gargle, Low-level laser therapy	2
**3**	67	M	0	Tongue cancer	Cisplatin, Fluorouracil, Cetuximab	N	Lidocaine gargle	2
**4**	38	F	0	Acute myeloid leukemia	Allotransplantation	N	Morphine hydrochloride hydrate, lidocaine gargle	3
**5**	60	F	0	Appendix cancer	Panitumumab	N	Salcote® capsule	2
**6**	80	M	0	Renal cell cancer	Pembrolizumab	N	Dexartin® oral ointment	2
**7**	71	M	1	Pharyngeal cancer	Cisplatin	70 Gy	N	2
**8**	38	F	2	Acute myeloid leukemia	Cyclophosphamide	N	N	2
**9**	55	F	0	Stomach cancer	Ramucirumab, paclitaxel	N	Lidocaine gargle, triamcinolone acetonide	2
**10**	68	F	0	Adult T-cell leukemia	Fludarabine phosphate, Busulfan	Total Body Radiation (2 Gy)	Dexartin® oral ointment, lidocaine	2

*F* female, *M* male, *N* not treated, *PS* performance status

At baseline, nine patients had Grade 2, and one patient had Grade 3 OM according to the NCI-CTCAE Version3.0 (medical examination findings). The details of concomitant analgesic use are shown in Table 2.

### Pain NRS (Primary outcome)

**Fig. 2:**
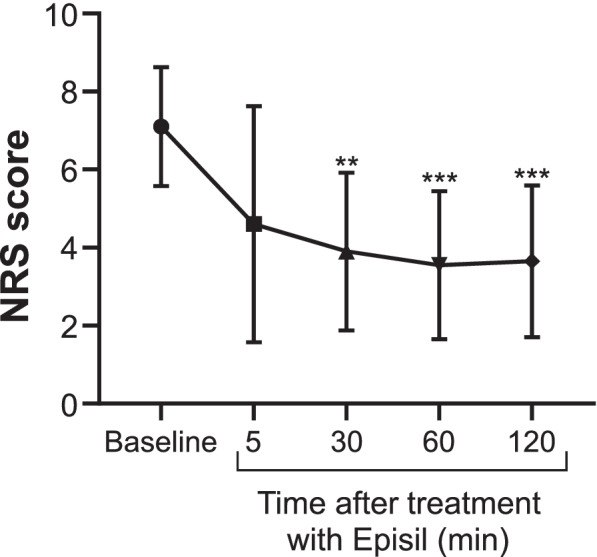
Changes in the numerical rating scale (NRS) scores over time**.** The NRS score of oral pain evaluation immediately before using Episil® (baseline) and 5, 30, 60, and 120 min after its application. The graph shows data representing the mean ± standard deviation values. NRS scores tend to decrease over time. There are significant differences in the oral pain score between baseline and 30, 60, and 120 min after application. **p ≤ 0.01, ***p ≤ 0.001

The mean pain score at baseline was 7.1 ± 1.4. The NRS score of oral pain decreased over time to 4.6 ± 2.87 at 5 min (*p* = 0.264), 3.9 ± 1.920 at 10 min (*p* = 0.0036), 3.55 ± 1.795 (*p* = 0.0004) at 60 min, and 3.65 ± 1.844 at 120 min (*p* = 0.0009) after the application of Episil® (Fig. 2). No patient used rescue analgesics during this period.

### Perception

**Fig. 3:**
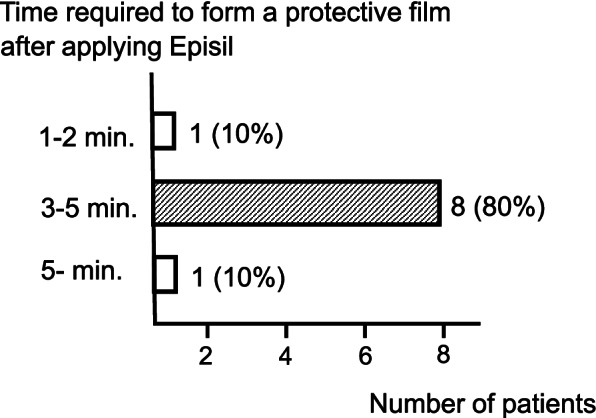
Time required to form a protective film after applying Episil®. In ninety percent of patients, Episil® formes a protective film within 5 min after application

Eighty percent of the patients reported that Episil® formed a protective film within 3–5 min after application, 1-2 min (10%), and 5-min (10%). (Fig. 3).

Regarding the usability of Episil®, 70% of patients thought it was easy to use. None answered “difficult” (Table 3), 80% felt “ordinary” in their mouth after Episil® application, and none felt “bad” (Table 3).

**Table 3:** Perception of Episil®

	Time after Episil® treatment (min.)				
	Baseline	5	30	60	120
Conversational difficulties					
None—barely any	2 (20%)	—	—	—	5 (50%)
A little troublesome	6 (60%)	—	—	—	5 (50%)
Very troublesome	2 (20%)	—	—	—	0 (0%)
Eating difficulties					
None—barely any	1 (10%)	—	—	—	5 (50%)
A little troublesome	5 (50%)	—	—	—	3 (30%)
Very troublesome	4 (40%)	—	—	—	2 (20%)
Ease of use of Episil®					
Easy to use	—	7 (70%)	—	—	—
A little difficult to use	—	3 (30%)	—	—	—
Difficult to use	—	0 (0%)	—	—	—
Feeling and comfort in the mouth when Episil® is used					
Good	—	2 (20%)	—	—	—
Ordinary	—	8 (80%)	—	—	—
Bad	—	0 (0%)	—	—	—
Changes in taste					
None—barely any	—	6 (60%)	10 (100%)	9 (90%)	8 (80%)
A little troublesome	—	4 (40%)	0 (0%)	1 (10%)	2 (20%)
Very troublesome	—	0 (0%)	0 (0%)	0 (0%)	0 (0%)
Stimulation of the mucous membrane					
None—barely any	—	8 (80%)	9 (90%)	10 (100%)	9 (90%)
A little troublesome	—	2 (20%)	1 (10%)	0 (0%)	1 (10%)
Very troublesome	—	0 (0%)	0 (0%)	0 (0%)	0 (0%)
Uncomfortable feeling					
None—barely any	—	6 (60%)	8 (80%)	8 (80%)	9 (90%)
A little troublesome	—	3 (30%)	2 (20%)	2 (20%)	1 (10%)
Very troublesome	—	1 (10%)	0 (0%)	0 (0%)	0 (0%)
Acceptance and willingness to continue using Episil®					
Want to keep using after this study	—	10 (100%)	—	—	10 (100%)
Don't want to use it anymore	—	0 (0%)	—	—	0 (0%)

—: not tested

During the treatment course after Episil® application, most patients felt no change in taste at 30 min (100%), 60 min (90%), and 120 min (80%) (Table 3). Regarding the mucous membrane stimulation, more than 90% of patients answered “no stimulation” or “none,” or if any, a slight uncomfortable feeling at any time point (Table 3).

### Oral-related functions

Oral-related functions consisted of two components as follows: speaking and eating. Speaking function at 120 min after Episil® application was improved compared to baseline. Two patients answered “very troublesome” before Episil® application; however, no patients answered “very troublesome” 120 min after application. The number of patients who answered “none – barely any” increased from 2 to 5 before and after Episil® application (Table 3). Improvement in eating function was similarly reported. The number of patients who complained of very troublesome eating was 4 before application, and it decreased to 2 at 120 min after Episil® application. Only one patient answered no difficulty in eating before application, and it increased to 5 at 120 min after application (Table 3).

Regarding the question about “Acceptance and willingness to continue using Episil®,” all patients answered that they “wanted to continue to use Episil® beyond the end of this study” (Table 3). The patient’s feeling of discomfort during Episil® use reported at the beginning of use did not significantly hinder the patients’ acceptance of the use of Episil®.

### Adverse events

During the observation period, no adverse events(Nausea, Vomiting, Others) or device failures were observed in any of the cases. Patients’ free comments on the use of Episil®; 1)it will be easier to use in form of mist, 2)the bottle is small and easy to carry, 3)the nozzle is hard to push, 4)the nozzle is too short; hence, it is difficult to deliver the contents to the affected area, 5)there is dripping from the top of the nozzle; thus, it is difficult to use, 6)Immediately after application, there was irritation; however, after a while, I became accustomed to it, and the pain disappeared, 7)Pain relief duration was shorter than 8 h, and it became painful in about 5 h, 8)The gel is tattered in the mouth, 9)Effective when used early on before oral mucositis becomes severe, 10)There is a sense of incongruity in the oral cavity, and 11)Episil® tastes too sweet for me, and it is quite uncomfortable. Many comments contained suggestions for improvements of the Episil® device.

## Discussion

The results of this study showed that Episil® is an effective device to relieve oral pain in Japanese patients with moderate to severe treatment-related OM, and its duration of action was determined to be long enough to support the patients’ oral intake. According to Hadjieva et al., who investigated the pain-relieving effect of Episil® in patients undergoing radiation therapy for head and neck cancer with OM, the mucositis pain score decreased rapidly 5 min after application, and this effect appeared to last for eight hours. It has been reported that even though the adhesive film gradually peels off over time due to abrasion, its effect is not totally diminished by a single meal [14]. The present findings are consistent with these results.

To alleviate the pain of OM associated with cancer treatment, in clinical practice, systemic administration of analgesics (e.g. acetaminophen, NSAIDs, or opioids) is prescribed according to the severity of the mucositis. Similarly, it has become common practice to apply a local anesthetic, such as lidocaine, directly to the pain site to reduce pain [18].

However, there have been some challenges with the conventional methods for alleviating the pain associated with OM. Administration of systemic analgesics is good for controlling resting pain; however, it is less effective for contact pain or movement pain during eating and speaking. Moreover, the therapeutic effect following the intake of these analgesics can be delayed. In addition, systemic administration of analgesics has adverse effects, such as renal dysfunction, NSAID-induced gastric mucosal disorder, and constipation and nausea due to opioid use, which might negatively affect the performance of the cancer treatment itself [19, 20]. The use of local anesthetics in patients with OM also has some problems. The effects of local anesthetics are immediate; however, their duration of action is not long, being approximately 20 to 30 min. Occasionally, anesthetics become ineffective during meals, and oral pain may reappear. Patients have to use local anesthetics multiple times in a day. Additionally, local anesthetics block all nerve activities; hence, they paralyze all sensations in the mouth and do not just numb the oral pain. Unfortunately, this feeling is far from necessary, and even aspiration may be a concern because due to impairing the smooth swallowing reflex. In the present study, Episil® showed a strong pain-relieving effect within a short time, its effects were long-lasting, and frequent use was unnecessary. According to the product information leaflet, the thickness of the adhesive protective film formed by Episil® is approximately 0.5 to 6.5 μm, there is almost no sense of incongruity, and the taste is hardly affected. Furthermore, it can be used without concerns about systemic side effects or altering oral sensation. This study was not an actual comparison with other treatments, such as local anesthetics; however, the abovementioned points suggest that Episil® does not interfere with cancer treatment or adversely affect dietary QOL. This may be a clinical advantage compared to systemic analgesics or local anesthetics.

Episil® has some major advantages as a pain relief formulation because, based on its mechanism of action, it does not elicit a pharmacological effect; rather, it simply offers physical wound protection. It is effective for breakthrough pain, such as contact pain during meals or talking, unlike systemic analgesics. Episil® neither causes discomfort nor disturbs the pleasure of eating, and its effect is immediate and persistent. Although systemic side effects and drug-drug interactions were not investigated in this study, Episil® does not contain any medicinal components; therefore, it is thought that there should be less concern about its side effects due to systemic interactions with other drugs. Thus, Episil® may safer as a supportive therapy during cancer treatment than other treatments, such as opioids or NSAIDs.

In particular, Episil® may also be valuable in patients with mild OM who have not yet been treated aggressively. The active management of mild OM cases is sometimes difficult. Although the patients had a slight tingling sensation or discomfort in their mouth, making eating slightly more difficult, it was not as painful as when local anesthetics or systemic analgesics were used. Therefore, in many cases, only oral care and simple gargling or no treatment at all had been provided for mild OM patients. This suggests that Episil® could be a new formulation to facilitate eating and drinking without causing discomfort to such mild OM patients and contribute to improving their QOL during cancer treatment.

The use of Episil® itself is simple, and its use relies on the patient’s self-management. Therefore, some precautions or considerations may be required for safer and more effective use. First, it takes little time for patients to gain experience in the use of Episil®. In the present study, the protective effects of Episil® were observed 3–5 min after its application. Thus, it seemed better to evaluate the effect after a while rather than immediately after application. Second, patients tended to apply more than the recommended quantities of Episil® into the oral cavity because it took some time for the effects of Episil® to manifest, and all patients wanted an immediate effect. However, excessive dosing may cause discomforts, such as nausea, vomiting, and even treatment interruption. Episil® is designed to drip enough to cover the entire oral cavity in a single press. The recommended dose is 1–3 pump strokes, starting with 1 pump stroke and applying more as needed. However, if the patient feels uncomfortable, it is important to limit the drip to approximately three times, even if they feel the drip is inadequate. Based on the recommended dosing, the patient should start with one pump stroke and apply more if needed. Third, some patients felt slight soreness or discomfort when applying Episil®. However, this slight soreness due to Episil®’s stimulation was usually within the allowable range and transient in nature. If patients feel strong stimulation after the application of Episil®, it can be managed by having the patients gargle with local anesthetics, such as lidocaine, before using Episil®. Fourth, Episil® should not simply be squirted, but also spread over painful mucous membranes using the optimal amount. Severe OM causes extensive and deep pain. It was difficult for most of the patients who cannot move their tongues because of severe OM to spread Episil® properly in their mouth. In such cases, it was necessary to spread the liquid using a safe alternative method, such as using a finger. In addition, severe OM disturbs proper oral cleaning and gargling, which causes the patient’s mouth to become filled with viscous, dirty saliva and results in worse oral hygiene. The viscous saliva in the mouth clings to the mucous membranes and prevents Episil® from effectively forming a protective film. In these patients who cannot receive proper oral cleaning and gargling, Episil® may not work effectively, and the patients may feel discomfort. In fact, they may feel strong discomfort. Thus, it is desirable to start Episil® at the stage of mild to moderate OM, before the symptoms become severe. Using Episil® at the early stage of OM, before severe OM occurs, makes it easier for patients to experience its actual efficacy. This may facilitate the patients’ continuous usage of Episil® effectively even if OM becomes more severe. Finally, an unhealthy oral cavity may cause local infections, increase the grade of OM, exacerbate pain, and prolong the time to heal. During immunosuppression by chemotherapy, local infections with OM cause a high risk of spreading and systemic infections, which is one of the major concerns. To control the risk of infection in the oral cavity, professional dental care should be provided by dental hygienists and dentists, and appropriate self-care instruction should be given. In the present study, all patients were provided with adequate basic oral care by a dental care team, and no patients developed oral infections during the study period. To safely use Episil®, it is important that professional dental care be continued to provide oral care to patients with OM.

Although it was not a direct feeling during use, several patients reported that the pump of the container was difficult to push down firmly; therefore, it was difficult to dose/squirt the contents in their mouths. Patients with poor general conditions may have difficulty applying Episil® by themselves and may require the assistance of a healthcare professional, their family, or others.

The study has several limitations. It was conducted as a preliminary study (the sample size was 10 casees), which makes difficult to indicate decisive conclusion. The modest sample size makes it difficult to draw decisive conclusions. This study doesn’t consider that the difference of the pathogenic mechanism of mucositis. Episil® has no medicinal properties and only physical protection of the ulcer, therefore we had thought that there was no difference in the effectiveness of Episil® depending on the pathogenic mechanism of mucositis. However, it cannot be denied that the effect of Episil® may differ depending on the pathophysiology of mucositis yet. In addition, the questionnaire used in this study was prepared with reference to the Camurus test (HS-05–160). As this test is not generally used in the clinical evaluation of OM, it is not easy to compare the results of this study to other studies previously reported. In assessing QOL in cancer patients, in particular, regarding the oral functions, a validated questionnaire on oral health may be more meaningful in further research[21].

We did not aim to compare Episil® with other treatments in this study, and we were not be able to set up an appropriate control arm in this study because there was no device that protects the mucous membranes, such as Episil®, had been approved for use in Japan. Since the mechanism of action is completely different between Episil® and other treatments, a simple comparison may be difficult; however, it will be necessary to continue investigation, exploring the synergistic effect of combined use.

Despite these limitations, Episil® seems to be effective in Japanese patients suffering from OM pain. Future research is warranted to explore Episil®’s efficacy.

## Conclusions

Episil® is effective for relieving pain in a rapid and long-acting manner in Japanese patients suffering from OM; it might help cancer treatment continue smoothly without interruptions, and it may improve the QOL of cancer patients.

## Data Availability

All the study related data has been included in the manuscript.

## Abbreviations

GDO: Glycerol dioleate
NCI-CTCAE: National Cancer Institute-Common Terminology Criteria for Adverse Events
NRS: Numerical rating scale
NSAID: Non-steroidal anti-inflammatory drugs
OM: Oral mucositis
QOL: Quality of life
SPC: Soybean phosphatidylcholine
